# Using a controlled stimulant to treat cocaine use disorder in the setting of attention deficit/hyperactivity disorder: a case study

**DOI:** 10.3389/fpsyt.2026.1740412

**Published:** 2026-01-21

**Authors:** Mack Bozman, Brent Collier, Aniket Malhotra, Gray McPherson, Li Li

**Affiliations:** Department of Psychiatry and Behavioral Neurobiology, University of Alabama at Birmingham, Birmingham, AL, United States

**Keywords:** cocaine craving scale, cocaine use disorder, dextroamphetamine-amphetamine, harm-reduction, sobriety

## Abstract

Cocaine Use Disorder (CUD), like all other stimulant use disorders, remains a challenging condition to treat, particularly in the absence of FDA-approved pharmacotherapies. While contingency management (CM) is considered first-line treatment and has demonstrated strong evidence for efficacy, it remains underutilized due to financial, philosophical, and logistical barriers. Off-label use of psychostimulants has emerged as a potential therapeutic strategy, especially in patients with comorbid attention-deficit/hyperactivity disorder (ADHD). We present a case of a middle-aged male with a long-standing history of ADHD and recent onset of CUD, who experienced frequent relapses despite multiple rehabilitation efforts. Following reinstatement of dextroamphetamine-amphetamine salts, the patient demonstrated sustained abstinence from cocaine for six and a half months at the time of this paper—his longest period of remission to date. His sobriety was supported by negative monthly urine drug screens and reduced cravings measured by the Cocaine Craving Scale (CCS). Current evidence supports CM and includes limited but growing support for stimulant substitution therapy. This case highlights the potential role of psychostimulants as a harm-reduction strategy in a carefully selected subset of patients with CUD and co-occurring ADHD. As stimulant use and overdose rates continue to rise, particularly with increasing adulteration by synthetic opioids, further research into effective, individualized treatments for stimulant use disorders, including CUD, is urgently needed.

## 1 Introduction

Stimulant use disorders (StUD) involve persistent use of stimulant drugs despite harmful consequences, often leading to neglect of rewarding, prosocial, or goal-directed activities. They represent a public health crisis associated with high rates of morbidity and mortality (1). Several other neurodevelopmental and psychiatric conditions increase the risk of StUDs including ADHD. Indeed, nearly a quarter of young adults with StUDs meet the DSM-5 TR Diagnostic criteria for ADHD (2). Individuals with ADHD also have earlier onset of StUDs, higher likelihood of polysubstance use, suicide rates, and relapse rates as well as more hospitalizations and lower treatment adherence (3).

StUDs include the use of Cocaine, amphetamine-related substances, and other stimulants with similar effects including dextroamphetamine, amphetamine, and methylphenidate, among others (1). Cocaine Use Disorder (CUD), along with other StUDs is notoriously difficult to treat, with frequent relapses, partly due to the lack of viable medication assisted treatments for this category of substance use (4).

The current first line intervention for CUD is contingency management (CM) which is a system in which patients are rewarded for meeting milestones such as negative urine drug screens, consecutive number of days maintaining sobriety, or attending therapy sessions (4). However, clinician and public opinion varies on utility and even ethics of this treatment modality (5). Despite the evidence in favor of using CM for treatment of all StUDs, it is not commonly used for these disorders. This is due to a variety of reasons, including lack of knowledge and unfamiliarity with CM, insufficient funds for CM incentives, and perceptions that CM is a form of “bribery” (5).

There are no FDA approved medical treatments for StUDs, another factor that makes treatment of these groups of disorders challenging. There have been many proposed treatments, including acupuncture, antidepressants, dopamine agonists, antipsychotics, anticonvulsants, disulfiram, opioid agonists, N-Acetylcysteine, and psychostimulants, though in a meta-analysis of available studies these treatments were not found to be effective (6). Although research on treating StUDs with psychostimulants is limited, this approach may be beneficial in selected patient populations (4). Cocaine remains the second leading cause of illicit drug overdoses in the U.S., with rising rates of stimulant use and increasing contamination of cocaine with fentanyl (2, 7, 8). The substance use epidemic demands harm reduction strategies and greater awareness of contingency management (CM) (7).

Here we will discuss potential treatment options as well as a review of the current data and a case presentation of a 43-year-old male patient diagnosed with ADHD as a child who later in life developed a cocaine use disorder. He failed multiple rehabilitation treatments, narcotics anonymous meetings, and family interventions until the treatment with Dextroamphetamine-amphetamine salts was initiated.

## 2 Case presentation

The Institutional Review Board (IRB) approval was waivered at the University of Alabama at Birmingham Hospital. Our case study focuses on a 43-year-old male who was diagnosed with ADHD in childhood. He had been prescribed dextroamphetamine-amphetamine salts, IR, 20mg, three times a day, for most of his adolescent and adult life and was stable on this until two years ago when he first tried cocaine at a social event. He reported using one to two grams of cocaine daily via insufflation. He reports he still took the dextroamphetamine-amphetamine salts a few times per week, though his primary stimulant at this time was cocaine. After this initial use, the patient was unable to remain in remission from cocaine use for more than two weeks at a time, despite attending multiple rehabilitation programs, support groups, and Narcotics Anonymous meetings. He had been diagnosed with comorbid Major Depressive Disorder (MDD) with anxiety and had been prescribed an antidepressant/anxiolytic regimen of citalopram, propranolol, and gabapentin, though he had only been taking gabapentin regularly. His primary psychiatrist eventually referred him to a Dual Diagnosis clinic. The patient also had a history of occasional cannabis use. His use of cocaine caused significant damage to his social, emotional, and occupational functioning.

Given the patient’s history of tolerating and responding well to dextroamphetamine-amphetamine salts, IR, for ADHD, it was decided that he would restart this medication for both ADHD and as a potential treatment for CUD. Following initiation, the patient was required to repeat urine drug screens (UDS) monthly at each clinical visit. The UDS result was positive for cocaine prior to the initiation but remained negative on all subsequent tests.

**Figure 1:**
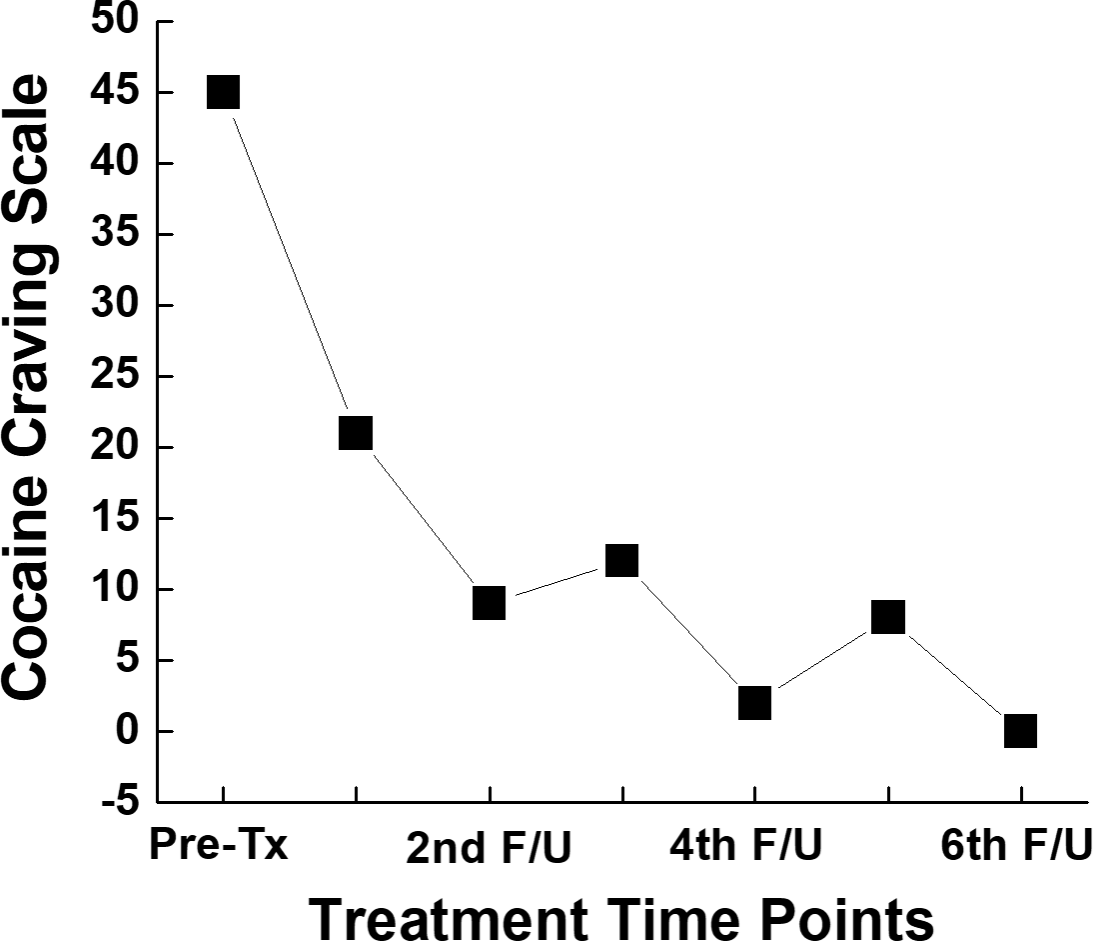
Graphical representation of total value of the five item self-reported CCS.

At each visit, the patient completed the Cocaine Craving Scale (CCS) (9), a validated self-report questionnaire used to assess current craving intensity. The initial CCS score was 45, the highest possible score (**Figure 1**). Dextroamphetamine-amphetamine salts, IR, were started at 10 mg twice daily and titrated to 20 mg three times daily over the course of 4 months. The final regimen reflecting the dosage he had been prescribed in the past as an adult for ADHD. His CCS scores decreased over time. His compliance with medication was monitored using the state Prescription Drug Monitoring Program, with no concerns noted. At the end of 4 months’ treatment, ADHD symptoms improved significantly, and his mood was stable. In addition, he planned to open a new company, was able to reconnect with his ex-wife and discuss their relationship, and restored the damaged relationship with his parents. He believes he has found himself again.

## 3 Discussion and conclusion

CUD and other StUD remain notoriously difficult to manage due to high relapse rates and persistent stigma. While the American Society of Addiction Medicine (ASAM) and the American Academy of Addiction Psychiatry (AAAP) identify CM as the first-line treatment for StUD (10). However, barriers such as lack of awareness, limited funding, and philosophical objections continue to hinder its adoption (6, 11). Second-line treatments include psychostimulants, though only contingency management programs were significantly associated with an increased likelihood of having a negative test result for the presence of cocaine (OR, 2.13; 95% CI, 1.62-2.80) (8). Use of psychostimulants for this purpose remains off-label. Both ASAM and AAAP emphasize the importance of identifying and managing comorbid conditions such as ADHD, depression, anxiety, and other substance use disorders (10).

A comprehensive workup—including a biopsychosocial assessment—should be completed, with a low threshold for conducting an electrocardiogram and creatine kinase testing, given the elevated risk for cardiac and renal complications in this population (10, 12). Use of psychostimulants as a treatment option for CUD may be viable, though there are many barriers to this treatment including cost/availability of psychostimulants, limited but promising research on this treatment option, potential risk of diversion and misuse of prescription psychostimulants, as well as stigma on the use of stimulants (7). Careful patient selection is essential, taking into account medical comorbidities such as prior history of ADHD with adequate response to stimulants family history including relatives with history of substance use disorders as well as with history of psychiatric conditions such as ADHD, depression, anxiety, or substance-induced psychotic or mood disorders. Additional factors such as housing stability is important as not all housing programs or care facilities may be equipped to prescribe controlled substances and/or the patient may or may not be able to afford certain stimulant medications. Finally, ensuring that the patient will have access to these medications is crucial as loss of access to stimulants may lead to relapse.

Despite fluctuation in craving scores, our patient has remained abstinent from cocaine—with the exception of a single relapse—for over six months following initiation of dextroamphetamine-amphetamine salts IR treatment. This represents his longest period of remission since first using cocaine. Due to the nature of a case report, our findings could not be generalized, and more cases are warranted to support our results. Our findings are also limited to concurrent ADHD and CUD, thus it is not clear if it could be applied to CUD only. However, combining our observation with previous reports (13), both regular release and extended release of amphetamine are promising to manage patients with comorbid ADHD and CUD. As global rates of substance use and co-occurring mental illness rise, identifying and expanding effective treatment options, including psychostimulants, becomes increasingly vital. Despite barriers stated above and heterogeneous results in the literature (14), further clinical trials, especially randomized clinical trials will be critical to provide solid evidence to support the use of psychostimulants in treating StUD, including CUD, with or without other psychiatric comorbidities.

## Data Availability

The original contributions presented in the study are included in the article/supplementary material. Further inquiries can be directed to the corresponding author.
